# Preferences for return of germline genome sequencing results for cancer patients and their genetic relatives in a research setting

**DOI:** 10.1038/s41431-022-01069-y

**Published:** 2022-03-11

**Authors:** Megan C. Best, Phyllis Butow, Jacqueline Savard, Chris Jacobs, Nicole Bartley, Grace Davies, Christine E. Napier, Mandy L. Ballinger, David M. Thomas, Barbara Biesecker, Katherine M. Tucker, Ilona Juraskova, Bettina Meiser, Timothy Schlub, Ainsley J. Newson

**Affiliations:** 1 Faculty of Science, University of Sydney, Sydney, NSW Australia; 2 Institute for Ethics and Society, University of Notre Dame Australia, Sydney, WA Australia; 3 School of Medicine, Deakin University, Geelong, VIC Australia; 4 Graduate School of Health, University of Technology, Sydney, NSW Australia; 5 Cancer Division, Garvan Institute of Medical Research, Sydney, NSW Australia; 6 Triangle Institute, Washington, DC USA; 7 Hereditary Cancer Centre, Prince of Wales Hospital, Sydney, NSW Australia; 8 Psychosocial Research Group, University of NSW, Sydney, NSW Australia; 9 Faculty of Medicine and Health, University of Sydney, Sydney, NSW Australia

**Keywords:** Cancer genetics, Human behaviour

## Abstract

Germline genome sequencing (GS) holds great promise for cancer prevention by identifying cancer risk and guiding prevention strategies, however research evidence is mixed regarding patient preferences for receiving GS results. The aim of this study was to discern preferences for return of results by cancer patients who have actually undergone GS. We conducted a mixed methods study with a cohort of cancer probands (*n* = 335) and their genetic relatives (*n* = 199) undergoing GS in a research setting. Both groups completed surveys when giving consent. A subset of participants (*n* = 40) completed semi-structured interviews. A significantly higher percentage of probands thought people would like to be informed about genetic conditions for which there is *prevention* or *treatment* that can change cancer risk compared to conditions for which there is *no prevention* or *treatment* (93% [311] versus 65% [216]; *p* < 0.001). Similar results were obtained for relatives (91% [180] versus 61% [121]; *p* < 0.001). Themes identified in the analysis of interviews were: (1) Recognised benefits of GS, (2) Balancing benefits with risks, (3) Uncertain results are perceived as unhelpful and (4) Competing obligations. While utility was an important discriminator in what was seen as valuable for this cohort, there was a variety of responses. In view of varied participant preferences regarding return of results, it is important to ensure patient understanding of test validity and identify individual choices at the time of consent to GS. The nature and value of the information, and a contextual understanding of researcher obligations should guide result return.

## Introduction

Germline genome sequencing (GS) is increasingly used to identify cancer risk and guide prevention [1]. While GS provides hope for reducing cancer morbidity and mortality, it also introduces ethical and implementation challenges [2].

GS can identify cancer variants that: (a) allow preventative intervention (clinically actionable), (b) have no preventative intervention available (non-actionable), although they can influence decisions relating to lifestyle, reproductive choices etc, (c) are of uncertain significance (VUS) or (d) are secondary to the diagnostic intention of testing (secondary findings). Pathogenic variants identified through patients’ GS will also have relevance to patients’ genetic relatives.

Research evidence is mixed regarding cancer patient preferences for receiving GS results. While some participants have reported wanting to know all results [3], others have declined testing, anticipating predictive knowledge as burdensome [4, 5]. Family concerns (particularly for parents), a previous cancer diagnosis and level of genomic knowledge are known to influence patient preferences [6].

Actionability of results has long been recognised as a criterion for returning GS results. Previous studies suggest that patients consider results with *personal* utility (such as for reproductive purposes) to be ‘actionable’, while researchers typically consider those with *clinical* utility to be ‘actionable’ [7, 8] and therefore worthy of return [9–11]. Research also suggests that a person’s preferences may be impacted by psychological factors, such as knowledge, worry about genetic risks [5], having genetically related children [12] and genetic causal beliefs [13], so that healthcare providers and patients may view the same results differently. These findings have implications for counselling prior to testing to manage patient expectations as well as for deciding which results to produce and return. It is therefore of great importance to understand how patients decide which results they wish to receive.

GS is still expensive in the community (~USD4000 on commencement of this study), and it is known that many patients who are interested in testing do not pursue it due to cost [14]. Adult GS is not publicly funded in Australia. To discern preferences for return of results by patients who are actually undergoing GS and to understand why they made this choice, we took the opportunity to assess preferences for return of results by patients with likely familial cancer and their genetic relatives who are actually undergoing GS as part of a research study, as these preferences are not studied outside of the hypothetical setting.

## Subjects and methods

### Participants

Participants were recruited to the Genetic Cancer Risk in the Young Study (RisC), which is being conducted at the Garvan Institute of Medical Research in Sydney, Australia. The primary aim of RisC is to identify clinically actionable, pathogenic gene variants that likely contribute to the development of cancer at an early age. RisC is recruiting probands with a likely genetic basis for their cancer diagnosis, and their genetic relatives, to undertake GS as part of the research protocol. The target population consists of [1]: adults with histologically confirmed malignancy under 40 years at diagnosis; OR having >1 primary cancer diagnosed <50 years; OR having >2 primary cancers at any age [2]; genetic relatives of RisC participants. All participants need to be able to read and speak English.

While testing is not initiated for clinical purposes, those participants found to carry a pathogenic cancer gene variant are referred to a genetic counsellor and offered a tailored risk-management plan through a subsequent study.

The Psychosocial Issues in Genomics in Oncology (PiGeOn) Project is a longitudinal, mixed methods sub-study of RisC, which aims to examine the psychosocial, behavioural and ethical impact of GS in this cohort. Patients consent to this study and the parent study at the same time. The consent process involved a verbal explanation of the study by a researcher, accompanied by written information. A subset of relatives gave consent with written information only (see Supplementary 1). The protocol for PiGeOn has been previously published [15]. A previous paper has also reported motivations for participation [16]. Both the RisC and PiGeOn studies were approved by the St Vincent’s Hospital Human Research Ethics Committee (HREC/16/SVH/24).

### Procedure

All participants were asked to complete a survey at baseline, described elsewhere [15]. In brief, four measures assessed which genetic conditions participants considered ‘people’ would like to be informed about, for example known genetic conditions caused by single/multiple genes, and whether there was prevention or treatment available to alter risk. Demographic/disease details and consent data were collected by the parent study. Consent data consisted of participant decisions regarding which results they elected to receive in the actual RisC study. Options offered were: ‘gene variant that causes cancer’; and/or ‘incidental [secondary] finding that may be important to my health’. These options referred to cancer and non-cancer genes, respectively, both of which were assessed in the American College of Medical Genetics (ACMG) gene list.

For the qualitative sub-study, a subgroup of participants was asked to participate in semi-structured interviews after giving consent to GS, but before any genetic information was available. Purposive sampling was used to ensure heterogeneity in the demographics and cancer-related characteristics of the sample. Telephone interviews were scheduled within 1–2 weeks of consent. Interviews were conducted by one researcher (NB) and continued until data saturation. Interview questions are found in Supplementary 2.

### Analysis

Descriptive statistical analysis of quantitative data was undertaken using IBM SPSS Statistics Version 25. Analysis of demographic/disease variables potentially associated with the desire to receive each type of result was performed using four multiple logistic regressions. The outcome variable was dichotomised as ‘yes, interested in receiving the result’, as indicated by participants’ ‘Yes’ response, vs. ‘not interested or unsure’, including ‘No’, ‘Maybe’, or ‘Don’t Know’. The predictor variables investigated included age; sex; education; urban versus rural place of residence; whether English was spoken in the home (proxy for culturally and linguistically diverse background); medical/science occupation; time since diagnosis; cancer incidence; and whether participants have first-degree genetic relatives with cancer diagnosis.

Interviews were recorded and transcribed verbatim. Thematic analysis [17] was used to code qualitative data. An initial coding tree was established and applied to further transcripts. New codes were developed iteratively as required and used to develop themes. Themes were developed and discussed by the research team, which comprised experts in medicine, psychology, genetic counselling, and bioethics. Differences were resolved through discussion, allowing rigour and reflexivity in the analysis. Triangulation of data was achieved through comparison of qualitative and quantitative results, and proband and relatives’ data, by framework analysis [18].

## Results

The current analysis includes the first 534 survey respondents at baseline. Probands (*n* = 335) had a mean age of 41.7 years with 66% being female. Relatives (*n* = 199) had a mean age of 63.0 years with 59% being female. All probands and 24% of relatives had a previous cancer diagnosis. The survey response rate was 92% for both probands and relatives, and 100% for interviews (*n* = 40). See Table 1.

**Table 1:** Demographics.

Demographic variables	Probands (*n* = 335)	Interviews (*n* = 20)	Relatives (n = 199)	Interviews (*n* = 20)
Sex (*n*, %)				
Female	220 (66%)	13 (65%)	118 (59%)	11 (55%)
Age (years)				
Median (IQR)	39 (15)	42 (15)	64 (11)	63(9)
Mean (SD)	41.72 (13.75)	46.10 (12.55)	63.04 (8.43)	62.65 (6.66)
Range	16–83	32–78	31–87	49–74
Education (*n*, %)				
Primary School	0	0	1 (0.5%)	0
Year 7 or 8	2 (0.6%)	0	9 (5%)	0
Year 9 or 10	23 (7%)	1 (5%)	38 (19%)	3 (15%)
Year 11 or 12	40 (12%)	2 (10%)	17 (9%)	1 (5%)
Vocational training	53 (16%)	4 (20%)	40 (20%)	2 (10%)
University—did not graduate	29 (9%)	1 (5%)	13 (7%)	1 (5%)
University—graduated	187 (56%)	10 (50%)	79 (40%)	12 (60%)
Unknown	1 (0.3%)	2 (10%)	2 (1%)	1 (5%)
Medical/science occupation				
(*n*, %)	27 (8%)	3 (15%)	16 (8%)	1 (5%)
Culturally and				
Linguistically Diverse (CALD) (*n*, %)^a^	74 (22%)	3 (15%)	18 (9%)	2 (10%)
Accessibility and Remoteness Index of Australia (ARIA) (*n*, %)				
Urban	314 (94%)	17 (85%)	168 (84%)	19 (95%)
Genetic children (*n*, %)	175 (52%)	17 (85%)	197 (99%)	20 (100%)
Cancer diagnosis (*n*, %)	335 (100%)	20 (100%)	48 (24%)	4 (20%)
Time since probands’ cancer diagnosis (years)				
Mean (standard deviation)	7.47 (9.39)	12.81 (12.47)	4.51 (5.29)	3.39 (2.55)
Range	0–52.17	0.83–41.83	0.08–35.30	0.83–9.20
First-degree relative diagnosed with cancer (*n*, %)	164 (49%)	18 (90%)	199 (100%)	20 (100%)

^a^Culturally and Linguistically Diverse (CALD) status identified by whether English is spoken in the home.

### Quantitative results

With regard to their own GS results, almost all participants elected to be informed about gene variants that cause cancer (97% of probands and 96% of relatives), secondary findings deemed to be important to their health (96% of probands and relatives) and in the event of their death, for information important to health to be made known to relevant health professionals involved in their or other family members’ care (98% of probands and relatives).

With regard to the survey results, significantly more participants (311 (93%) probands and 180 (91%) relatives) thought people would like to be informed about genetic conditions for which there is *prevention* or *treatment* that can change risk versus *no prevention* or *treatment* (216 (65%) probands and 121 (61%) relatives), a difference of 28% [*p* < 0.001] for probands and 30% [*p* < 0.001] for relatives (Table 2). There was more uncertainty (‘Maybe’ or ‘Don’t know’ responses) amongst participants when thinking about whether people would like to be informed about genetic conditions for which there is no prevention or treatment (103 probands and 69 relatives) versus availability of prevention or treatment that can change risk (21 probands and 19 relatives).

**Table 2:** Survey results: What sort of genetic conditions do you think people would like to be informed about when they take part in genetic research?.

	Probands, *n* (%)	Relatives, *n* (%)
Known genetic conditions caused by one gene, for which there is *no prevention* (e.g. diet, exercise) or *treatment* that can *change the risk* (e.g. inherited blindness)		
Yes	216 (65)	121 (61)
No	13 (4)	9 (5)
Maybe	73 (22)	40 (20)
Don’t know	30 (9)	29 (15)
Known genetic conditions caused by one gene, for which there is *prevention* (e.g. screening) or *treatment* that can *change the risk* (e.g. breast or bowel cancer)		
Yes	311 (93)	180 (91)
No	0	0
Maybe	6 (2)	11 (6)
Don’t know	15 (5)	8 (4)
Known genetic conditions caused by many genes, which can have a major impact on health, for which there is *treatment as well as lifestyle factors* (e.g. diet, exercise, stopping smoking) which can *modify the risk* (e.g. cancer, heart disease)		
Yes	303 (90)	183 (92)
No	0	1 (0.5)
Maybe	15 (5)	7 (4)
Don’t know	14 (4)	8 (4)
Known genetic conditions caused by many genes, which usually have a lower impact on health, for which there is *treatment as well as lifestyle factors* which can *modify the risk* (e.g. asthma)		
Yes	285 (85)	174 (87)
No	2 (0.6)	4 (2)
Maybe	27 (8)	13 (7)
Don’t know	18 (5)	8 (4)

More participants (303 (90%) probands and 183 (92%) relatives) thought people would like to be informed about genetic conditions for which there is treatment and lifestyle factors that can have a *major impact* on health versus a *lower impact* on health (285 (85%) probands and 174 (87%) relatives), a difference of 5% (*p* = 0.001) for probands and 5% (*p* = 0.012) for relatives (Table 2).

Multiple logistic regression demonstrated that, amongst probands, having a higher educational attainment and an English-speaking background were associated with thinking that people would desire to be informed about genetic conditions caused by one gene, for which there *is prevention* or *treatment*. For each category increase in education, odds increased by a factor of 1.39 (*p* = 0.038) for probands and 1.58 (*p* = 0.004) for relatives, and the odds of English-speaking probands considering these results desirable were 4.02 times that of probands from a non-English-speaking background (*p* = 0.005) (Supplementary Table 3).

Similarly, the odds of English-speaking probands thinking that people would wish to receive results about genetic conditions caused by *multiple genes* which could have a *major impact* on health for which prevention or treatment were available, were 2.41 times that of probands from a non English-speaking background (*p* = 0.041). Relatives with higher education were also more likely to consider these results desirable, with odds increasing by a factor of 1.59 (*p* = 0.007) per category increase in education (Supplementary Table 4), and also results about genetic conditions caused by many genes, which can have a *lower impact* on health: for every category increase in relatives’ education, the odds of considering that people would want to receive these results increased by a factor of 1.70 (*p* = 0.001) (Supplementary Table 5).

Conversely, probands with lower educational attainment were more likely to favour results about known genetic conditions caused by *one gene*, for which there is *no prevention* or *treatment* that can change risk. For every category increase in education level, the odds of thinking that people would like these results decreased by a factor of 0.77 (*p* = 0.006) (Supplementary Table 6). No other variables were significantly associated with the preference options listed in the survey.

### Qualitative results

Responses of probands and their genetic relatives were combined, as themes were shared between groups. While most participants were clear on their preferences at the time of interview, some could not remember which results they had elected to receive, while others had ongoing decisional uncertainty, continuing to ponder whether and which results they should receive. Themes identified in the transcripts were: (1) Recognised benefits of GS, (2) Balancing benefits with risks, (3) Uncertain results are perceived as unhelpful and (4) Competing obligations. Quotes to illustrate each theme can be found in Table 3.

**Table 3:** Participant quotes.

Theme	Participant quote
1. Recognised benefits of testing	
1a. Personal benefits	*It’s a little bit like providing a … scientific crystal ball, and it’s not going to necessarily be correct and it might not come to fruition, but it’s a bit like going to …a scientific psychic that goes, look, this is possibly in your path. And… maybe you could do something to avoid it. (Female proband, 41 years); Some people think I’m absolutely crazy for [being tested], and, all I can think about is how fortunate I am to be doing testing that may change my life… (Female proband, 42 years); I guess I decided that I would rather know and deal with the consequences as they come than not know and deal with the uncertainty of what I don’t know. (Male proband, 37 years); What’s the point of knowing things that you can’t do anything about? (Female relative, 53 years, no cancer)*
1b. Family benefits	*There is cancer down the line of my family… So I think for the future of all the grandchildren and all the other siblings in the family, it is a good idea for us… to try and maybe prevent... (Female proband, 42 years); I think the more information you have, the more potential benefit you have…even though we can’t act on everything right now, it doesn’t mean that [treatments] won’t be developed in the future. (Female proband, 32 years)*
1c. Community benefit	*The way I see it, [my daughter] has been helped by research that’s been done in the past. So, that needs to continue.... (Female relative, 67 years, no cancer)*
2. Balancing benefits with risks	*How do you stratify the information you get? If I’m at high risk of cancer and my kid is potentially at high risk of cancer, is that the most important thing, or is it the fact that I might have exercise-induced asthma? (Female proband, 37 years); I think some people would really get stressed. It might be a time-bomb hanging over their heads, if they knew something was going to happen. (Female relative, 74 years, no cancer); The real issue, for me, is a policy issue. [The government] can legislate for information to be divulged—there may be all the safeguards in the world now, but if a law comes in which says … all insurance companies are entitled to know it [genomic test results], then that’s a risk. (Male proband, 57 years); I’ll know about [my results] and I might decide then whether I’d tell anyone else in the family, but I mightn’t tell them if it’s going to affect their life insurance of something. (Male relative, 71 years, no cancer)*.
3. Uncertain results are perceived as unhelpful	*I think, research is so amazing, it’s just going to get better and better and at some point, hopefully… people can say, now we understand what this means and that hopefully will benefit someone. (Female proband, 40 years); I wouldn’t be very worried because… I’ve already got cancer. What really is there that would make a massive difference? (Female proband, 40 years); I don’t know that it would be helpful for the person. That would worry them, they’d just go through life thinking, yes, there’s something wrong, but we’re not sure what it is. (Male proband, 56 years)*
4. Competing obligations	*It’s the future generation you are talking about, the children and the grandchildren. (Male relative, 69 years, no cancer); It should be a law, if they find they can help future generations, and then people will be forced to. (Male relative, 69 years, no cancer); That’s a really hard question. I mean, when we talk about life-saving information, this is not as clear cut as… when someone is bleeding out from a car accident. (Male proband, 37 years); Feed it back to their general practitioner…[who can then] suggest, ‘Hey, you want a heart test?’ (Female proband, 43 years); If the genome shows that I’m going to get dementia, well maybe I’d rather not know, at my stage of life…Maybe my thoughts would have been different if I was 50 years younger, I don’t know.’ (Female relative, 74 years, no cancer)*

### Recognised benefits of GS

This cohort, who had already given consent to germline GS, were enthusiastic about the benefits of testing with regards to all categories of results. Reflecting the quantitative results, interest in this information increased according to its perceived usefulness—whether it represented clinical, personal or individual utility (through answering questions about family cancers). Benefits were recognised from personal, family and community perspectives.

#### Personal benefits

Many participants wanted to receive all available results as a means to prevent future disease. As such, many participants felt that being tested through the research study was a privilege, since GS is not publicly funded in Australia. GS was also viewed as a way to reduce uncertainty about individual risk. Because the value of GS lay in its perceived utility, some participants did not want to receive results that were non-actionable.

#### Family benefits

Relatives, most of whom were parents, were particularly focussed on family reasons for participating in GS testing. Relatives had joined the study at the invitation of the proband (who was diagnosed with cancer). Probands were also aware of the familial implications and considered possible ways future generations could avoid disease. All positive results were therefore valued, even if the (potential) full benefit lay in the future.

#### Community benefit

GS results were also seen as contributing to the common good. Many participants were aware of the benefits they or their children had received from previous scientific research.

### Balancing benefits with risks

Despite awareness of the benefits, the choice to undergo GS within the research study was seen as a balancing act between risks and benefits. Some participants had concerns about being overwhelmed by too much information. They desired information to be presented in an understandable framework, with accessible language.

The emotional cost of receiving difficult information was also recognised, with varying abilities of individuals to cope with knowledge of increased disease risk.

The most frequently articulated risk referred to increasing (individually risk rated) insurance premiums for products such as life insurance, although this interpretation was often erroneous, as all probands and some relatives would likely be declined such insurance anyway, due to their cancer diagnosis. Additionally, Australian insurers do not access health records directly but instead act on information disclosed by applicants and there is no obligation for individuals to disclose a relative’s genetic test result. (Health insurance is community risk rated in Australia).

In view of these risks, some participants envisaged themselves as gatekeepers, where they would calculate the risk/benefit score, and decide whether to notify relatives on these grounds.

### Uncertain results are perceived as unhelpful

Participants were asked to imagine how they would feel if they received a VUS result (which was not an option for this study). Responses to the question varied, ranging from incomprehension (‘*I don’t understand what you mean, either you are or aren’t at risk.’—Male relative, 59 years, no cancer)*, through to confidence of future certainty and clinical utility.

Participants with current cancer felt that their disease made a VUS irrelevant. The uncertainty integral to the experience of their disease trajectory meant that they did not feel that anything could significantly worsen their situation.

Due to the lack of perceived utility, many participants were not interested in receiving VUS results. Some participants thought it could actually be harmful as it could increase uncertainty, yet this was based on the flawed presumption that a VUS was necessarily pathogenic.

### Competing obligations

Participants were asked whether they thought it was appropriate to participate in genetic research but choose not to receive results. Responses depended on whether participants prioritised individual or communal perspectives. Some participants accepted this scenario readily, generally attributing such a view to either altruism (participating solely to advance science), or the emotional inability to cope with challenging news. Others condemned this attitude in view of the potential of results to improve others’ health.

On being asked whether a choice not to receive results should be overridden if an actionable result was found, most participants hesitated. While a minority accepted the right of the individual to make such a choice, whatever its basis, most were reluctant to miss an opportunity to provide information of clinical utility that could have a significant health impact. Some respondents saw the return of such results as obligatory (and as such should be enforced by law) due to the perceived potential community benefit, warning of future legal action if disease developed as a result. However, the current limits of genomic science and subsequent complexity of the situation was also acknowledged.

Others suggested giving such a patient a further opportunity to deliberate and decide, or passing the results to a healthcare provider who could discuss it with the patient, such as by “*dropping a few hints*”. Some relatives felt that their age negated the need to be forewarned about potential disease.

## Discussion

This study explored the views of cancer patients and their genetic relatives regarding which GS results they thought should be returned in the research context. We found that most participants expected that people would want to receive any results that they perceived to be useful (interpreted as actionable) and related to significant disease. The decision to receive results was calculated using a risk/benefit analysis, most thinking that benefits outweighed the risks, as has been found in previous research with cancer patients in the research setting [19].

The utility of genomic information was valued even if it was only potential future utility, as reported previously [20]. While utility is an accepted value by which to determine which results to return in the research setting, debate is ongoing over whether it is clinical utility, personal utility or individual utility that should be the standard [21]. The current cohort expressed willingness to receive results that had both personal (health related) and clinical utility. As such, if it is clinically indicated, practically feasible and if the patient agrees, it may be appropriate to return results of both personal and clinical utility. This could be said to respect patient best interests, as well as promote public support of genomic research [22].

It is noteworthy that a small number of respondents did not want any results returned. To put these responses in context, this was a research study and relatives, parents in particular, reported that their primary motivation was to help their children [16]. Most participants assumed such an attitude reflected an altruistic motivation for research participation. Previous studies have found that research participants who elected not to receive genomic findings may already have sufficient genomic information from previous testing or felt that information would not change their personal healthcare decision-making [23]. Some relatives in our cohort thought they were too old to worry about predictive GS results.

A number of interviewees were keen to be told all available information, assuming that receiving any kind of information will reduce uncertainty. This is common in both research and clinical contexts [23, 24]. There is no doubt that societal discourse has conditioned us to believe that information is good, as it implies control over our health. The rhetorical power of potential ‘life-saving’ GS encourages many patients to seek maximum information, and patients undergoing GS have reported a sense of empowerment [25], although high value placed by patients on GS has previously also been associated with overestimation of its validity [25]. However, in this context (research, where resources may be more limited) it is pertinent to ask whether it is appropriate to ask if patients want all results in view of the resources required to investigate all findings and report them, particularly if some results are of unknown significance, or if there is no plan to continue updating results once the study has concluded. At least, care needs to be taken during the consent process to manage patient expectations, especially due to the importance of the healthcare provider in influencing patient decisions to be tested [26]. Despite the support for the return of actionable secondary findings [9–11, 27], return of VUS and non-actionable secondary findings in translational research remains controversial, with debate in the literature about whether testing (and reporting) should be limited to the diagnostic question or whether there is an obligation to also screen for potentially clinically significant findings [28].

Actionable results were seen as more desirable by participants with greater educational attainment and who spoke English as their primary language. This could be related to better access to information, increased understanding of the significance of heightened cancer risk, and greater self-efficacy to act on results in these groups [29], and mirrors earlier findings that understanding predicts return of result preferences in cancer patients [5]. Numerous studies in the general population have found that genetic knowledge is lower amongst those with less education and low health literacy [30, 31], which could in turn impact choices regarding result return. One finding in variance to this trend was the greater perceived interest in non-actionable results seen in probands with lower educational attainment. We contend that this could be due to their misunderstanding of the limited utility of non-actionable results, therefore being more likely to believe these results would be valued by others. It will be important to recognise population needs and differences when implementing GS clinically, given that it is operating against a background where many from culturally and linguistically diverse groups are underserved in genomics provision (and indeed in the databases that support interpretation of results) [32]. Those who speak a language other than that being used in the study (or in clinic) are likely to need aids to facilitate understanding.

No other variables were associated with result preference, which is contrary to earlier studies [5, 12, 13] which identified a range of demographic and psychological predictors of preferences. For example, Guo et al. [12] and Kaphingst et al. [5] found that young women with breast cancer who had genetically related children were interested in carrier status regardless of actionability, whereas this variable was not associated with any result return preference in the current study. Our study included older participants (up to age 83) which may have impacted worry about children, although the majority of our sample were younger (median of 39 years). Forty-four percent of our sample were men however, and it is possible that men have a different perspective on their children’s future health risks than women.

Like earlier studies [5], we found no impact of clinical factors (such as time since diagnosis) on preferences. Kaphingst et al. [5] hypothesised that this may be because participants considered genomic results more informative regarding their general health than their cancer risk, and thus their cancer characteristics were not relevant to genomic decision-making. Indeed, these authors found that participants had diverse motivations for undertaking GS and learning their results, often unrelated to cancer [33]. While our participants frequently referred to cancer risk in interviews, this was generally with reference to the future, for either themselves or their children. Thus, their current cancer may have had less impact on their thinking and decision-making than their concerns about future risk.

There were mixed responses regarding the right of the individual to ‘not know’ their personal results from the study, even though they may benefit other patients in the future (through research). Clinically, genetics professionals have expressed support for the option for patients to decline to receive results [27], a position supported by the ACMG [34] as well as the National Pathology Accreditation Advisory Council in Australia [35]. While the choice to not engage with genetic conditions is well documented [36], there is some concern about potential implications, for both health professionals and other family members, of not passing on relevant information. The “right not to know” may, some argue, be better framed as a preference than a right; and may not hold in all circumstances [37]. Indeed, when the information could have critical health significance, the majority of respondents in this study supported overruling of individual choice. However, per our above comments, this view may be based on an at-present premature belief that genomic information can be life-saving. While the debate on the right not to know (especially in a research context) continues, it seems prudent to adopt a cautious approach.

Some interviewees were interested in receiving VUS results on the grounds that they may be of future interest. As our understanding of the medical significance of genetic data continues to evolve rapidly, so the implications of findings can change over time [38]. Developments in therapy may also alter treatment recommendations for genetically linked disease [39]. In the clinical context it has been shown that in 10–16% of VUS, explanatory variants were discovered through re-analysis 1–3 years later [28]. It is possible that there is a new role for a healthcare provider to take responsibility to regularly check for revised interpretations of variants. Certainly, annual reviews are now occurring in some laboratories [40]. Yet, apart from the difficulty in maintaining current contact information when the testing is done in a research context, this presents significant practical and ethical challenges. For example, concerns have been raised over ‘diverting the information deluge from physicians to patients’ [41]. It could also be argued there is no duty to recontact, as even clinicians do not have the responsibility for monitoring all aspects of patient health on an ongoing basis [42]. Limiting how long the obligation to return results should last for research projects has long been supported [43]. Guidance in this area is still emerging [44, 45]. In the clinical context, the genetic counselling appointment provides an opportunity to discuss the arrangements for review, for example patients might request referral to a cancer genetics service every 3–5 years.

Dynamic consent platforms have also been suggested as a way to coordinate and streamline long-term access to updated GS results [46]. The ongoing problem of primary physicians being under-skilled for interpreting genomic results [47] remains problematic if this role is given to them, as would be the resources required.

Some participants who did not want to receive their results personally were willing to have any relevant information passed on to their relatives or their health professionals. Thus reluctance to engage with results may reflect inability to cope with results. Those returning GS results need to check participant preferences to avoid communicating unwanted information, but also to ensure that relatives, who may benefit from learning the results, are not disadvantaged [48]. However, in view of differing interpretations of what makes results valuable to the individual, healthcare professionals should take care to clarify patient expectations prior to testing taking place, in both research and clinical contexts. This could include a staged return of results [49].

Limitations to this study include the inability to study the survey differences observed in preferences of those with lower education or from a non-English-speaking background in the interviews due to inadequate sample size. This study included a qualitative component that is not meant to be generalisable, and GS was conducted in a research setting. Other cohorts may respond differently.

## Conclusion

The current study found that most participants in a research setting wished to receive GS information. While utility was an important discriminator in what was seen as valuable for this cohort, there were a variety of interpretations and responses regarding what types of results should be returned. In view of diverse opinions, it is important to identify individual choices and manage expectations during the GS consent process. However, as the perceived utility of GS can diminish after receiving results, [50] and in view of the known challenges in ensuring fully informed consent [20], we suggest that the nature and value of the information for both patients and their genetic relatives and a contextual understanding of researcher obligations, should guide GS result return in the research context.

## Supplementary information

Supplementary Information

## Data Availability

Additional data are available from the corresponding author on reasonable request.
