# Supplementary material for: Preferences for return of germline genome sequencing results for cancer patients and their genetic relatives in a research setting
Source: Eur J Hum Genet. 2022 Mar 11;30(8):930–7. doi: 10.1038/s41431-022-01069-y (PMC9349221; doi:10.1038/s41431-022-01069-y)
Supplement: Supplementary file 1 — Supplementary Information [file 41431_2022_1069_MOESM1_ESM.pdf]

Supplementary Information

Supplement 1: Genetic Cancer Risk in the Young Study Participant Information Sheet and Consent Form (modified)

Genetic Cancer Risk in the Young Study
Participant Information Sheet

1. Introduction

You are invited to take part in this research project. This is because you or a family member have had cancer. You will be asked to donate a sample of blood which will be used for genetic research.

This Participant Information Sheet/Consent Form tells you about the research project. It explains the tests and treatments involved. Knowing what is involved will help you decide if you want to take part in the research.

Please read this information carefully. Ask questions about anything that you don't understand or want to know more about. Before deciding whether or not to take part, you might want to talk about it with a relative, friend or your local doctor.

Participation in this research is voluntary. If you don't wish to take part, you don't have to. You will receive the best possible care whether or not you take part.

If you decide you want to take part in the research project, you will be asked to sign the consent section. By signing it you are telling us that you:
• Understand what you have read
• Consent to take part in the research project
• Consent to have the tests and treatments that are described
• Consent to the use of your personal and health information as described.

Before you decide whether or not you wish to participate in this study, it is important for you to understand why the research is being done and what it will involve. Please take the time to read the following information carefully and discuss it with others if you wish.

2. What is genetic research?

Genes are made of DNA – the chemical structure carrying your genetic information that determines many human characteristics such as the colour of your eyes or hair. Researchers study genes in order to understand why some people have a certain condition such as cancer and why some people do not. Our genes are like a set of instructions that help bodies to work properly. We all have approximately 20 000 genes. This is called our genome. Some of our genome is unique to each of us, but we do share most of it with our relatives.

Cancer is caused by alterations (variants) in our genes that make cells grow out of control and spread abnormally. Variation in our genomes is normal. Some variants can increase a risk of developing certain diseases (such as cancer), while others (such as variants that

determine eye colour) do not. Some, but not all, genetic variants that increase the risk of developing cancer are inherited (passed on from parent to child) and have implications for all blood relatives. We already know about some of these, but there is still much more to learn.

Whole genome sequencing
Until now we have only been able to look for inherited variants in a few genes. For example we know that breast cancer is sometimes caused by a variant in the BRCA1 or BRCA2 gene. Recent developments in science and technology mean that it has become possible to look at the whole of a person's genome at once, to try to understand more about the genetic causes for disease. In this study we are planning to see whether there are any gene variants in all 20 000 of your genes, using a technique called whole genome sequencing.

Whole genome sequencing involves obtaining the sequence of all of your 20 000 genes in one test. When your sequence is examined, results may show that a gene is normal, or show a variant thought to cause disease, or a variant that has unknown significance. Some of this information might be relevant to your or your family's risk of developing cancer, but there is a small chance that whole genome sequencing may detect a variant in your genome that is not related to your risk of cancer. This is called an 'incidental finding'. It's also important to remember that if nothing is found, this does not rule out a genetic contribution to your cancer.

In this study, we plan to offer you information about your risk of cancer and information about some other conditions if we are sure about the medical significance of the finding. We do not plan to offer you information if it is of uncertain significance. It is up to you to decide how much of your information you would like to be told. Some people like to know this information, while others prefer not to be told. It may help to think in advance about what you might do with any information you may find out.

3. What is the purpose of this research?
The aim of the study is to understand more about the genetic variants that contribute to inherited cancer. This is important for people with cancer and their families. We hope that this knowledge will lead to more personalised cancer treatments with better outcomes, improved cancer screening, increased options for reducing cancer risks and more fully informed lifestyle and reproductive decisions. We aim to also assess health related costs.

In this study we would also like to understand what people think and feel about this type of genetic testing.

7. What are the possible benefits of taking part?
You will be contacted if the testing shows important information about you, and you will be asked if you wish to know the results. The results may be important to you as they may provide:

- Information about risk of an inherited condition
- Information that might influence a decision to have children
- Information that might affect your ability to obtain insurance or employment.

In addition, if the testing shows important information about your relatives contact with your relatives about the testing is encouraged. You may wish to do this yourself or ask the researchers to contact them on your behalf.

Should you inform your relatives and they wish to know your results, genetic counselling will be arranged by the Study co-ordinator to explain what the results mean for you and to support you as necessary.

It will be necessary to refer you for re-testing by genetic services outside this research project. There may be some costs associated with re-testing that will not be covered by the study and will have to be covered by you, if you choose to go ahead.

8. What are the possible risks and disadvantages of taking part?
Genetic testing involves the study of genetic material (typically DNA) that is shared with your blood relatives. Genetic research is undertaken for many reasons, including discovering more accurate ways of predicting disease within a group of people, or in people where there is strong family history or predisposition of disease.

Genetic testing may raise important issues. Although few may be expected to arise, your awareness of this is important for you to think about and carefully consider before agreeing to participate. Genetic information may have implications for you and your blood relatives.

Learning about the results from genetic research might affect you and your family emotionally. In some cases, the result may give certainty that you do not have a disease but could also create uncertainty or be upsetting; if for instance, the test indicates an increased risk of developing a disease which has no known prevention, treatment or cure.

It is important to understand that results from genetic research will usually not indicate that you have a disease or disorder, or whether you will develop it. Research may only show that you have an increased **risk** of developing a disease or disorder. Even then, there is no guarantee that you will develop the condition or any indication of the likely age you might get the disease or how serious the disease might be.

You may learn information from your test result about inherited diseases or disorders that may affect others, such as your brothers or sisters. This could interfere with family relationships. You may be faced with the decision to make the family aware of the existence of genetic information. Family members may or may not wish to know this information.

Any research results that could be of significance to you or your family will need to have the tests repeated and the results verified. This will involve having a blood sample taken and having it retested in an accredited testing laboratory. This is standard practice for all patients receiving the results of genetic testing. There may be some charges to you. Counselling may be provided free of charge if it is appropriate. Before a test is repeated to verify a research finding, you will be informed about the possible risks involved for you. This is especially important for individuals who are found to have a genetic mutation that is associated with an increased risk of developing a disease such as cancer or heart disease.

- **You may have a cancer gene variant**

There is a chance that you have a gene variant that makes you more likely to develop cancer. Other members of your immediate family and other blood relatives may also have the same gene variant. This gene variant could be passed on to the next generation. If you do have a gene variant, you and possibly other family members may have a higher risk of developing cancer, but we do not know exactly how high this risk might be. At this time there is no treatment for these gene variants.

In the consent form we will ask you to indicate if you wish to be informed about variants in cancer genes. In the future we may ask you to confirm your decision. You can change your mind at any time. If you have a cancer gene variant and you have indicated that you wish to be informed, we will send you a letter inviting you to visit a Family Cancer Clinic to see a specialist doctor or genetic counsellor. In the meantime, we will also give you the contact details of a genetic counsellor that you may contact any time during business hours. At the Family Cancer Clinic, the genetics specialist can help you to think about what this might mean for you and your family, describe any processes for confirmatory genetic testing and discuss the screening and risk management options that may be available and support you as you learn about the gene variant. If you are worried about a family history of cancer, you can ask to be referred to the Family Cancer Clinic before the results of the research become available. Your GP can refer you to a local clinic.

- **You may have a gene variant that is not related to cancer**

There is a chance that whole genome sequencing may detect a gene variant that is not related to cancer. This is called an incidental finding. Incidental findings can sometimes be important to your future health and the health of your family (blood relatives) and future children. The findings can include gene variants that cause or may mean an increased risk of a serious health condition. These health problems may include but not be limited to heart conditions, dementia, high blood pressure and high cholesterol.

In the consent form we will ask you to indicate if you wish to be informed about incidental findings that may be important to your health and the health of your blood relatives. You may choose whether or not to be informed. In the future we may ask you to confirm your decision. You can change your mind at any time. If you choose to be informed we will send you a letter advising you that we have detected a genetic variant that may be important to your health. If you choose to learn more, we will arrange an appointment with an appropriate clinical specialist to explain more to you about what the genetic variant could mean for you and your family.

- **If you have a gene variant there may be insurance implications**

Statutory or contractual duties may require you to disclose results of genetic tests or analysis to third parties (for example, insurance companies, employers, financial and educational institutions), particularly where results provide information about health prospects. If the results of your genetic tests are not available to you or you choose not to have the results given to you, then your future requests for insurance may not be affected by participating in this research.

Details of your family history are relevant in assessing your risk profile for certain forms of insurance. The ability to obtain private health insurance is not changed by your family history, genetic test results or health status. The Financial Services Council (FSC) has a voluntary agreement within its membership that any existing life, trauma or disability insurance that you may have will not be affected by your participation in the study.

Genetic information actually acquired by you as a result of your participation in this research may have implications for you (or your relatives') ability to obtain cover for certain risk rated insurance products offered either alone or as part of a superannuation product (eg insurance products providing cover for: life disability (income protection), trauma or any business or bank loans which require a policy for life (disability or trauma) and may impact upon the amount you pay for and scope of protection provided by such products.

- **You may experience some psychological distress**

You may feel that some of the questions we ask are stressful or upsetting. If you do not wish to answer a question, you may skip it and go to the next question, or you may stop immediately. If you have a gene variant, support will be provided by a Family Cancer Clinic.

- **Genetic testing may reveal unexpected misattributed paternity or maternity**

In some testing situations, if a person's social parent is not their biological parent, genetic testing might reveal it. This can happen when certain types of tests are done on several members of the one family. If this is detected, the study will not disclose it to you unless required by law to do so, which is unlikely.

- **There is a small risk to your privacy due to stored data**

There is a small risk to your privacy because personal information is used in the record linkage process. We will supply your personal information (name, date of birth, address) to the relevant registry or databank so that they can identify you correctly. The registry will retrieve all the relevant associated information and send it back to the study. All safety measures have been put in place to ensure the confidentiality of your information. Strict guidelines regarding data transfer, storage and access will be adhered to.

We wish to link data with cancer registries and the Australian Institute of Health and Welfare to confirm cancer diagnoses and obtain other health related information.

9. What will happen to my test samples?

Your blood sample will be used for the purposes of this research project. Genetic material (DNA and RNA) will be extracted and sometimes long living cell lines will be established.

Your blood sample will be stored in a re-identifiable manner so that if we find any genetic results that may be significant to the health of you or your family we will be able to give you that information should you wish to learn about it.

Any tumour samples collected from pathology services will also be stored in a re-identifiable manner. All your samples will be stored indefinitely.

10. What is the potential impact on my family if I take part?

We will ask you to give us health information about your relatives. Any information you give us will be kept confidential. We will invite some of your first degree relatives to participate in the study. We will ask you to get their permission before contacting them. We will not contact your relatives without your permission. If the research discloses that one of your family members may be at risk of a life-threatening or serious illness for which treatment is available or pending, this information may, with the prior approval of a Human Research

Ethics Committee, be offered by the study doctor to the family member, even if you as the participant do not consent to this.

11. Will I be given the results of this research project?

Your genetic test results important to health will be available to you should you wish. It is your decision whether you wish to be informed of these results. Before you decide if you wish to have your genetic test results, it is important that you read the information above regarding risks carefully so that you can make an informed decision and see a genetic counsellor if you so wish.

If we find a gene variant and you have indicated that you want to be informed about this information, we will send you a letter asking you to come to a familial cancer centre or another medical centre as needed. We will also give you the contact details of a genetic counsellor that you may contact any time during business hours. The genetic counsellor will be able to answer your questions and help you arrange your visit to the familial cancer centre.

Genetic information is complex and can be influenced by other factors including environment and lifestyle. Because genetic information is complex and sensitive, the results should be discussed with a clinical geneticist and genetic counsellor who can give you details that are relevant to you, answer your questions and discuss your concerns.

In the future, if during the course of this research project we discover new information that is important for your health care, you will be asked whether you wish to receive the results (this may require you to have the test repeated in a clinical laboratory). If you agree, we may contact you if such a situation arises.

Genetic Cancer Risk in the Young Study

Consent Form

Declaration by Participant

I wish to be informed if I am found to have a gene variant that causes cancer

YES ☐ NO ☐

I wish to be informed if I am found to have an incidental finding that may be important to my health

YES ☐ NO ☐

In the event of my death, any information important to health may be made known to relevant health professionals involved in my care and the care of other family members

YES ☐ NO ☐

In the event of my death, the information important to health may be made known to:

Name ___________________________________ Relationship _______________________

Contact details ____

Supplement 2: Interview Schedule.

1. Do you think you should receive all the information found in your test, or just the information relevant to your treatment?
2. How do you think you would feel if you were told that changes in your genes had been found, but that the meaning of this change was uncertain?
3. What do you think makes genetic information valuable to people?
4. Have you thought about whether this blood test would be of interest to your relatives? Do you plan to talk to your relatives about your test and the results?
5. Do you think it's okay to participate in this kind of research but choose not to receive any results at all? Or is it okay for people to be given genetic information, say if it could lead to cancer treatment, even if they said they didn't want to receive it?

Supplementary Table 3. Multiple logistic regression for thinking people would like to be informed about 'Known genetic conditions caused by one gene, for which there is *prevention* (e.g. screening) or *treatment* that can *change the risk* (e.g. breast or bowel cancer)' (yes vs no/maybe/don't know)

	**Probands**		**Relatives**	
Independent variable	Odds ratio (95% CI)	*p* value	Odds ratio (95% CI)	*p* value
**Sex**				
Female	0.94 (0.35-2.52)	0.899	1.25 (0.45-3.53)	0.671
Male	Ref.		Ref.	
**Age (yrs)**	1.01 (0.96-1.06)	0.807	0.97 (0.01-1.05)	0.465
**Education**	1.39 (1.02-1.91)	0.038*	1.58 (1.16-2.16)	0.004**
**Medical-science occupation**	‡		‡	
Yes				
No				
**English-speaking background**				
Yes	4.02 (1.52-10.65)	0.005**	‡	
No	Ref.			
**ARIA**				
Urban	0.59 (0.06-5.45)	0.639	1.03 (0.27-3.97)	0.965
Remote/Rural	Ref.		Ref.	
**Biological children**				
Yes	0.89 (0.30-2.63)	0.828		
No	Ref.			
**Cancer diagnosis**				
Yes			1.27 (0.33-4.91)	0.724
No			Ref.	
**Time since probands' diagnosis (years)**	0.99 (0.94-1.06)	0.847	1.06 (0.95-1.19)	0.314
**Family member diagnosed with cancer**				
Yes	1.45 (0.56-3.80)	0.448		

No	Ref.	

*** $p < .001$ | ** $p < .01$ | *$p < .05$ |

Ref. = Reference category

‡ Removed from the model due to insufficient variability in responses

Supplementary Table 4. Multiple logistic regression for thinking people would like to be informed about 'Known genetic conditions caused by many genes, which can have a major impact on health, for which there is *treatment as well as lifestyle factors* (e.g. diet, exercise, stopping smoking) which can *modify the risk* (e.g. cancer, heart disease)' (Yes vs No/Maybe/Don't Know)

	**Probands**		**Relatives**	
Independent variable	Odds ratio (95% CI)	*p* value	Odds ratio (95% CI)	*p* value
**Sex**				
Female	1.01 (0.43-2.34)	0.988	1.79 (0.61-5.31)	0.291
Male	Ref.		Ref.	
**Age (yrs)**	1.02 (0.97-1.06)	0.473	0.99 (0.91-1.07)	0.708
**Education**	0.97 (0.72-1.31)	0.843	1.59 (1.14-2.22)	0.007**
**Medical-science occupation**				
Yes	0.74 (0.20-2.77)	0.656	‡	
No	Ref.			
**English-speaking background**				
Yes	2.41 (1.04-5.60)	0.041*	‡	
No	Ref.			
**ARIA**				
Urban	1.05 (0.22-5.10)	0.954	1.78 (0.50-6.27)	0.373
Remote/Rural	Ref.		Ref.	
**Biological children**				
Yes	0.50 (0.19-1.29)	0.152		
No	Ref.			
**Cancer diagnosis**				
Yes			2.55 (0.46-14.22)	0.286
No			Ref.	
**Time since probands' diagnosis (years)**	0.98 (0.93-1.03)	0.397	1.08 (0.94-1.23)	0.285
**Family member diagnosed with cancer**				

Yes	1.72 (0.76-3.90)	0.197	
No	Ref.		

*** $p < .001$| ** $p < .01$ | *$p < .05$

Ref. = Reference category

‡ Removed from the model due to insufficient variability in responses

Supplementary Table 5. Multiple logistic regression for thinking people would like to be informed about 'Known genetic conditions caused by many genes, which usually have a lower impact on health, for which there is treatment as well as lifestyle factors which can modify the risk (e.g. asthma)' (Yes vs No/Maybe/Don't Know)

Independent variable	Probands		Relatives	
	Odds ratio (95% CI)	*p* value	Odds ratio (95% CI)	*p* value
**Sex**				
Female	1.08 (0.55-2.12)	0.823	1.73 (0.66-4.55)	0.265
Male	Ref.		Ref.	
**Age (yrs)**	1.00 (0.97-1.04)	0.894	0.99 (0.92-1.06)	0.722
**Education**	1.03 (0.81-1.29)	0.833	1.70 (1.24-2.33)	0.001**
**Medical-science occupation**				
Yes	1.32 (0.37-4.74)	0.671	0.33 (0.06-1.93)	0.217
No	Ref.		Ref.	
**English-speaking background**				
Yes	2.00 (0.98-4.08)	0.058	2.01 (0.36-11.17)	0.427
No	Ref.		Ref.	
**ARIA**				
Urban	1.44 (0.44-4.73)	0.552	2.73 (0.94-7.99)	0.066
Remote/Rural	Ref.		Ref.	
**Biological children**				
Yes	0.53 (0.25-1.13)	0.10		
No	Ref.			
**Cancer diagnosis**				
Yes			2.38 (0.56-10.05)	0.238
No			Ref.	
**Time since probands' diagnosis (years)**	1.00 (0.96-1.04)	0.930		

**Family member diagnosed with cancer**		
Yes	1.13 (0.59-2.18)	0.708
No	Ref.	

*** $p < .001$ | ** $p < .01$ | *$p < .05$

Ref. = Reference category

Supplementary Table 6. Multiple logistic regression for thinking people would like to be informed about 'known genetic conditions caused by one gene, for which there is *no prevention* (e.g. diet, exercise) or *treatment* that can *change the risk* (e.g. inherited blindness)' (Yes vs No/Maybe/Don't Know)

	**Probands**		**Relatives**	
Independent variable	Odds ratio (95% CI)	*p* value	Odds ratio (95% CI)	*p* value
**Sex**				
Female	0.73 (0.44-1.22)	0.233	0.89 (0.48-1.65)	0.710
Male	Ref.		Ref.	
**Age (yrs)**	1.00 (0.98-1.03)	0.801	1.00 (0.96-1.03)	0.862
**Education**	0.77 (0.63-0.93)	0.006**	1.19 (0.99-1.43)	0.066
**Medical-science occupation**				
Yes	1.10 (0.48-2.54)	0.819	0.66 (0.22-1.97)	0.457
No	Ref.		Ref.	
**English-speaking background**				
Yes	1.31 (0.74-2.31)	0.361	1.14 (0.38-3.45)	0.812
No	Ref.		Ref.	
**ARIA**				
Urban	1.13 (0.42-3.04)	0.807	1.06 (0.47-2.40)	0.891
Remote/Rural	Ref.		Ref.	
**Biological children**				
Yes	1.32 (0.75-2.32)	0.341		
No	Ref.			
**Cancer diagnosis**				
Yes			0.71 (0.36-1.43)	0.340
No			Ref.	
**Time since probands' diagnosis (years)**	0.98 (0.95-1.01)	0.229	1.01 (0.96-1.07)	0.672

**Family member diagnosed with cancer**			
Yes	0.77 (0.48-1.26)	0.300	
No	Ref.		

Ref. = Reference category
